# Supplementary material for: Music listening and stress recovery in healthy individuals: A systematic review with meta-analysis of experimental studies
Source: PLoS One. 2022 Jun 17;17(6):e0270031. doi: 10.1371/journal.pone.0270031 (PMC9205498; doi:10.1371/journal.pone.0270031)
Supplement: Attachment — Submitted filename: Review_PONE-D-21-22806.pdf [file pone.0270031.s002.pdf]

This systematic review and meta-analysis examined the effects of music listening after experimentally induced stress on stress recovery in healthy participants. The authors found no evidence for a cumulative effect of music listening on stress recovery. They did find that the effectiveness of music was moderated by several factors, including musical genre, type of music selection, musical tempo, and type of stress recovery outcome, although definite conclusions on the nature of these effects could not be drawn.

The study addresses a very timely question within the growing body of research on music and stress, and is well-executed. As such, it provides a valuable and much-needed contribution to the research field. The selection of study parameters and moderators of interest is convincing, the results are presented in a clear and comprehensible way, and the authors provide a thoughtful interpretation of their findings, which they appropriately put into perspective by acknowledging the limitations of their study. Furthermore, they provide several helpful and well-considered recommendations for future research. The manuscript is well-written.

I only have several minor comments.

1. In discussing the potential moderating effect "Self- vs. experimenter selected" (page 9), the authors mention two presumed explanations for this effect, namely increasing perceived control and serving self-regularity goals. For a somewhat more comprehensive picture, it may be worth adding the potential roles of liking and familiarity as further mechanisms behind the suggested higher effectiveness of self-compared to experimenter-selected music in promoting stress recovery.

2. In the abstract (and throughout the theoretical sections of the paper), it is stated that participants of the studies included in the meta-analysis/review "*were either exposed to music or silence.*" I find this misleading, since in the Method section, it is stated on page 10 that to be included, *"studies should compare music listening to silence or a comparable auditory stimulus (e.g., white noise, audiobooks)".*

Apart from the fact that it is not evident in what sense and to what extent silence can be considered comparable to auditory control stimuli, the use of the label "silence" to capture all non-music control conditions, is confusing. Please adapt the instances where you currently refer to silence by using more accurate wording (e.g. "silence or an auditory control condition").

3. Page 11: "*When authors did not or could not provide the required information (e.g., due to data no longer being accessible), the outcome was dropped from the meta-analysis. Based on these criteria, the final sample for the systematic review consisted of 17 studies. Following attempts to obtain missing information, the final sample for the meta-analysis consisted of 14 studies.*"

This is phrased in a confusing way – it is not clear what the conceptual difference between these selection steps is. Please rephrase this in a way that makes it less confusing.

4. On page 11, the authors point out that "*Stress induction procedures in included studies were not always successful. Given that successful stress induction procedures are crucial to ensure that participants experience some physiological or psychological change they may recover from, in our moderator analysis we examined whether the effect of music listening on stress recovery differed based on the outcome of a study's stress induction check (manipulation check)".*

I fully agree with the authors that, for music to exert an effect on stress, a physiological and/or psychological stress response needs to be present, from which participants may then recover. I find it therefore difficult to understand why studies which failed to induce stress (i.e. did not report a successful stress induction) were included in the meta-analysis in the first place. The fact that the successfulness of the stress induction, surprisingly, did not affect the extent of stress recovery does not really resolve my concern.

Could the authors briefly comment on this issue, and motivate their decision to still include these studies in their meta-analysis (either under "stress induction checks" on page 11, or in the discussion section)?

5. Page 11: "*In our moderator analysis, we examined whether the effects of music listening on stress recovery were reliable across general (neuroendocrine, physiological, psychological) and specific outcome types*."

I am not sure whether the moderator analysis allows any claims about the *reliability* of the effects across outcome types. In theory, an effect could be highly reliable across many outcome types, while at the same time still being clearly stronger for some outcome types than for others (hence being moderated by them), right? Wouldn't it be more correct to state that it was assessed to what extent the size of the effect on stress recovery depended on outcome type (or some equivalent formulation)? I am no expert on this issue, but I invite the authors to reconsider their wording.

6. There is a type on page 15: Wisagreements → Disagreements

7. As the authors rightly point out on page 6, stress recovery involves a process in which "*changes that have occurred in response to a stressor revert to pre-stress baselines".* To quantify stress recovery, it therefore seems crucial to take individual pre-stress baseline levels into account.

To the reader, it does not readily become clear whether the effects derived from the studies included in the meta-analysis indeed reflect the extent to which stress levels "return to baseline". From Table 4, the included studies seem to be a mix of 1) studies reporting differences in change scores with respect to pre-stress baseline levels and 2) studies reporting raw group differences in post-music stress levels. This may require some sort of disclaimer.

Could you please reflect on these analytical differences and their (possible) implications for the interpretation of your meta-analysis, in relation to the term "recovery"?

8. On page 37-38 you write: "*Khalfa et al. [55] reported that post-stressor cortisol decreased more rapidly for participants who listened to experimenter-selected classical music, compared with participants who sat in silence"*

In Table 2 you write, when referring to this study*: "Increase in post-stressor cortisol for music group significantly lower compared to control group (+)".*

These descriptions differ – could you please adapt the main text to match the (correct) description in the table?

9. On page 40-41, you write: "*While previous reviews suggest that music-based interventions may be moderately beneficial for stress-related outcomes, particularly in medical and therapeutic settings, our results suggest that the magnitude of this effect for healthy individuals may be more modest."*

While I largely agree with the contents of this paragraph (and with the further comments on this issue on page 46), I think the term "healthy individuals" (to label the category for which music is less effective for stress recovery) does not capture the essence of the differences between the different types of studies, and hence using this term may be a bit misleading.

As is stated further down the paragraph, the stress in studies conducted in medical and therapeutic settings likely has a more protracted time course, which does not directly have to do with the participants being (not) healthy. Furthermore, stress may differ in intensity between laboratory and medical real-life/settings, and the effectiveness of music may depend on the research setting as well.

It would be great if you could somewhat adapt the wording of this paragraph, to avoid the impression that the (non-) effectiveness of music depends on the participants being healthy. Rather, it seems more likely that several (interrelated) factors associated with the different research *settings* (e.g. type, intensity and duration of stress) are driving these differences. You might e.g. use the term "healthy individuals under brief, experimentally induced stress" instead.
