# Supplementary material for: Music listening and stress recovery in healthy individuals: A systematic review with meta-analysis of experimental studies
Source: PLoS One. 2022 Jun 17;17(6):e0270031. doi: 10.1371/journal.pone.0270031 (PMC9205498; doi:10.1371/journal.pone.0270031)
Supplement: Attachment — Submitted filename: Review.docx [file pone.0270031.s003.docx]

Review:

The authors address an important research question as they aim at systemizing the empirical evidence on beneficial effects of music listening on stress recovery. Overall, the manuscript is well written and the authors demonstrate methodological rigour and diligence on many instances. However, I have some major concerns that question the adequacy of the hypothesis and statistical approach as well as the search strategy.

Major Concerns:

1) The authors identified 14 studies that are quite heterogenous in nature. I ask myself whether the approach of a meta-analysis is adequate for this rather small number of studies given this vast heterogenity. Furthermore, not all studies were successfull in stress induction. Wouldn't it be reasonable to exclude these studies from the analysis?
2) Concerning the search strategy, I wondered that PubMed was not included. Searching this data base might be useful as the number of studies identified is quite small.
3) Inclusion and exclusion criteria are not specifically justified. For example, in Figure 1 exclusion criteria are presented, e.g. 'no music presented after stressor'. This might be the reason why the study of Thoma et al. (2013) (<https://pubmed.ncbi.nlm.nih.gov/23940541/>) is not party of the review, although I consider it highly relevant in the context of music listening and stress reduction. Overall, I would describe and justify more in detail criteria for inclusion and exclusion of studies.
4) Introduction: first paragraph: I really like the introduction to the topic, as the aspect of stress in daily life is emphasized. I wondered why the authors did not include ambulatory assessment/ecological momentary assessment studies in their review, as these studies have high ecological validity. I recommend to expand the review and to assess independently the evidence concerning controlled studies with high internal validity on the one hand and daily life studies with high ecological validity on the other hand.
5) It may be my personal opinion, but I was irritated by the many instances the authors use the term 'beliefs', e.g. abstract 'given the popular and widespread belief'. My recommendation is to re-word this and acknowledge the empirical evidence underlying this statement.

Minor Concerns:

Abstract: 'beneficial for stress' should be specified (beneficial for stress reduction)

Abstract: Please report how many participants in total were included in the 14 studies

Abstract: please specify that (randomized)-controlled studies were included

Introduction: 'It is a popular and widespread opinion that music may be beneficial for

stress recovery [10]': I am not convinced that the citation is that adequate in this context. Levitin demonstrates in this book many instances for beneficial effects of music. I am not satisfied with labeling his statement as 'popular and widespread opinion'. Furthermore, I do not consider this book an optimal citation for this peer-reviewed journal article as there is a vast body of empirical evidence available.

Introduction l.60: My first thought was to question the necessity of this review given the fact that an extensive review was just published. Although, the authors justify their review in the ensuing paragraph, I would recommend to state immediately more clearly that the scope of the other review was different.

Introduction l.78: Why not include studies demonstrating effects on cortisol?

l.133: I would not recommend to write 'smaller amounts of salivary alpha-amylase'. Rather, less activity of alpha-amylase.

l.153: Study 54 refers to an ambulatory assessment study – therefore, there was no control to silence or noise in a comparable manner to experimental studies. Please re-word.

l.164: I do not agree with the statement that the candidate mechanism underlying beneficial effects of music has already been identified. I would rather prefer to see here a more comprehensive statement acknowledging that the exact underlying mechanisms remain to be elucidated and that different notions exist, e.g., literature by Koelsch…

l.274: IgA is named as outcome measure but has not been introduced. As it is an immune marker, the introduction should contain some information on interactions among stress and immune system.

l.310: some typos need revision

l.425: Can you please indicate the range of music duration? As there is literature available on the effects of different music durations on beneficial effects, I assume that the range was very limited among these studies. Therefore, I would not state that duration is not important. Rather, that the range in experimental studies is not vast enough to allow for meaningful comparisons.
