# Supplementary material for: Music listening and stress recovery in healthy individuals: A systematic review with meta-analysis of experimental studies
Source: PLoS One. 2022 Jun 17;17(6):e0270031. doi: 10.1371/journal.pone.0270031 (PMC9205498; doi:10.1371/journal.pone.0270031)
Supplement: Attachment — Submitted filename: ma20211025_rebuttal_PLOS_v1.docx [file pone.0270031.s004.docx]

Dear Prof. Dr. Nater,

Thank you for your e-mail in response to our submission to PLOS ONE [PONE-D-21-22806], in which you explained your decision concerning our manuscript, enclosing the reviewers’ comments. We have carefully addressed each reviewer’s comments and adjusted our manuscript accordingly. Our responses to each reviewer’s comments have been provided in a point-by-point manner below.

We hope the revised version of our manuscript will fully meet PLOS ONE’s rigorous publication criteria. We look forward to hearing from you.

Warm regards,

Krisna Adiasto, MSc.

On behalf of all authors.

**Response to Reviewer #1**

Dear Reviewer,

Thank you for taking the time to review our manuscript. We appreciate your kind words about our work. More importantly, we sincerely appreciate the constructive comments and suggestions you have provided for various portions of our review and have thus updated our manuscript to address them. Below, we provide a summary of the changes we have made. You will see your comments in bold, our responses in regular text, and excerpts from our manuscript in italics (unless otherwise indicated):

**1. In discussing the potential moderating effect “Self- vs. experimenter selected” (page 9), the authors mention two presumed explanations for this effect, namely increasing perceived control and serving self-regularity goals. For a somewhat more comprehensive picture, it may be worth adding the potential roles of liking and familiarity as further mechanisms behind the suggested higher effectiveness of self-compared to experimenter-selected music in promoting stress recovery.**

We agree that preference and familiarity potentially play a role in making self-selected music more beneficial compared to experimenter selected music for the purpose of stress recovery. As we strive to make our review as comprehensive as possible, we have added the following sentences on the roles of preference and familiarity in our discussion of the potential moderating effects of self- vs. experimenter selected music (page 9):

*“Furthermore, previous studies have found that listening to self-selected music may help elicit stronger and more positive emotional responses regardless of a song’s valence (positive or negative) and arousal (high or low), possibly due to increased preference and familiarity towards the self-selected music (Jiang et al., 2016; Pereira et al., 2011; Sharman & Dingle, 2015). In theory, self-selected music should thus be more beneficial compared to experimenter-selected music for the purpose of stress recovery.”*

And again in our discussion of the moderator analyses (page 45):

*“The comparison of musical features between self-selected and experimenter selected music may also offer a more nuanced perspective on the role of preference and familiarity. Specifically, preferences and familiarity towards certain songs could be described in terms of specific (combinations of) musical features. For example, an individual may prefer music with slow tempo, mellow timbre, and moderate loudness. This approach is often leveraged by music recommender systems, such as those implemented by music streaming platforms (e.g., Spotify, Deezer, Apple Music, etc.), with the goal of recommending songs that listeners are likely to engage with. Future studies could investigate the extent to which preference and familiarity might differ between self-selected and experimenter selected music with similar combinations of musical features, to further clarify the role of selection in the relationship between music listening and stress recovery.”*

Jiang, J., Rickson, D., & Jiang, C. (2016). The mechanism of music for reducing psychological stress: Music preference as a mediator. *The Arts in Psychotherapy, 48*, 62-68. https://doi.org/10.1016/j.aip.2016.02.002

Pereira, C. S., Teixeira, J., Figueiredo, P., Xavier, J., Castro, S. L., & Brattico, E. (2011). Music and emotions in the brain: Familiarity matters. *PLOS ONE, 6*(11), e27241. https://doi.org/10.1371/journal.pone.0027241

Sharman, L., & Dingle, G. A. (2015). Extreme metal music and anger processing. *Frontiers in Human Neuroscience, 9*, 272. https://doi.org/10.3389/fnhum.2015.00272

**2. In the abstract (and throughout the theoretical sections of the paper), it is stated that participants of the studies included in the meta-analysis/review “were either exposed to music or silence.” I find this misleading, since in the Method section, it is stated on page 10 that to be included, “studies should compare music listening to silence or a comparable auditory stimulus (e.g., white noise, audiobooks)”.**

**Apart from the fact that it is not evident in what sense and to what extent silence can be considered comparable to auditory control stimuli, the use of the label “silence” to capture all non-music control conditions, is confusing. Please adapt the instances where you currently refer to silence by using more accurate wording (e.g., “silence or an auditory control condition”).**

Thank you for your suggestion. It was not our intention to use the label ‘silence’ as an overall term for all control conditions across studies. When the change was justified (i.e., when the cited studies indeed had an auditory control condition), we have adjusted the instances in which use of the label ‘silence’ only was not accurate. For example, in the Abstract:

*“As such, to clarify the current literature, we conducted a systematic review with meta-analysis of randomized, controlled experimental studies investigating the effects of music listening on stress recovery in healthy individuals. In fourteen experimental studies, participants (N = 706) were first exposed to an acute laboratory stressor, following which they were either* ***exposed to music or a control condition****.”*

On page 4:

*“Indeed, studies on music listening and stress recovery in healthy individuals are equivocal: although music listening is considered beneficial for physiological stress recovery, several studies have reported no differences in heart rate, heart rate variability, respiration rate, or blood pressure recovery between participants who listened to music and those who either* ***sat in silence or listened to an auditory control*** *[18-21].”*

On page 7:

*“For example, music listening has been associated with lower heart rate [49-51], systolic blood pressure [22, 50, 52], skin conductance [18, 20, 53, 54], and cortisol [55]* ***compared to silence or an auditory control condition.****”*

*“Furthermore, studies have demonstrated that listening to music may influence mood [60, 61]. Indeed, music listening has been associated with lower negative affect [38], higher positive affect [19, 62], and fewer self-reported depressive symptoms [38]* ***compared to silence or an auditory control condition.****”*

On page 16:

*“In the present study, a Hedges’ g of zero indicates the effect of music listening on stress recovery is equivalent to* ***silence or an auditory control****. Conversely, a Hedges’ g greater than zero indicates the degree to which music listening is more effective than* ***control****, while a g less than zero indicates the degree to which music listening is less effective than* ***control****.”*

On page 22:

*“This estimate suggests that, taking all variations in music and outcomes into consideration, the effect of music listening and silence have equivalent effects on stress recovery is equivalent to* ***silence or an auditory control****.”*

**3. Page 11: “When authors did not or could not provide the required information (e.g., due to data no longer being accessible), the outcome was dropped from the meta-analysis. Based on these criteria, the final sample for the systematic review consisted of 17 studies. Following attempts to obtain missing information, the final sample for the meta-analysis consisted of 14 studies.”**

**This is phrased in a confusing way – it is not clear what the conceptual difference between these selection steps is. Please rephrase this in a way that makes it less confusing.**

Thank you for pointing this out. To make the distinction between the two steps clearer, we have rephrased the sentences in question to:

*“Based on these criteria, the final sample for the systematic review portion of our manuscript consisted of 17 studies. Finally, for studies to be included in the meta-analysis portion of our review, means and standard deviations of stress recovery outcomes following stressor cessation must be available. Corresponding authors were contacted when this information was not available. When authors did not or could not provide the required information (e.g., due to data no longer being accessible), the outcome was dropped from the meta-analysis. Thus, following attempts to obtain missing information, the final sample for the meta-analysis portion of our review consisted of 14 studies.”*

**4. On page 11, the authors point out that “Stress induction procedures in included studies were not always successful. Given that successful stress induction procedures are crucial to ensure that participants experience some physiological or psychological change they may recover from, in our moderator analysis we examined whether the effect of music listening on stress recovery differed based on the outcome of a study’s stress induction check (manipulation check)”.**

**I fully agree with the authors that, for music to exert an effect on stress, a physiological and/or psychological stress response needs to be present, from which participants may then recover. I find it therefore difficult to understand why studies which failed to induce stress (i.e., did not report a successful stress induction) were included in the meta-analysis in the first place. The fact that the successfulness of the stress induction, surprisingly, did not affect the extent of stress recovery does not really resolve my concern.**

**Could the authors briefly comment on this issue and motivate their decision to still include these studies in their meta-analysis (either under “stress induction checks” on page 11, or in the discussion section)?**

Thank you for mentioning this. We agree that it is particularly important to further address the inclusion of studies with unsuccessful stress induction checks, as you and another reviewer have put forward similar concerns on the matter.

In the previous version of our manuscript, three studies were coded to have ‘unsuccessful’ stress induction checks. One of these studies (Scheufele, 2000) was erroneously coded, as the author did report a marked increase in heart rate following their stress induction procedure compared to baseline. Meanwhile, in the remaining two studies (Gan et al., 2016 & Labbé et al., 2007), the authors have hinted that their respective stress induction procedures were successful. However, we coded these as ‘unsuccessful’ since the statistical analyses comparing post-stressor and baseline values of their stress recovery outcomes were missing. We ultimately decided to still include these two studies in the meta-analysis given that the reported mean scores for certain stress recovery outcomes still suggested there was an increase from baseline from which participants could recover from. For example, in Gan et al. (2016), mean state anxiety for their three conditions during the stress task were *m_sedative_* = 46.97, *m_stimulative_* = 50.51, and *m_control_* = 52.00, compared to baseline means scores of *m_sedative_* = 38.80, *m_stimulative_* = 38.09, and *m_control_* = 36.17.

Based on this, we have thus updated the results of our analysis for the “stress induction checks” moderator (page 27), to reflect the change in coding for Scheufele (2000):

***“Stress induction checks.*** *There were no significant differences in the effects of music listening on stress recovery for studies with successful (*g *= 0.17301, 95% CI [-0.2635, 0.6155],* p *= .399625) and unsuccessful (*g *= 0.062361, 95% CI [-0.0894, 0.201.66],* p *= .115355) stress induction checks,* β1 *= -0.108257,* p *= .525661.”*

Next, we have added a paragraph to acknowledge the inclusion of studies with unsuccessful stress tasks in our Discussion (page 49):

*“Two studies with less successful stress induction procedures were still included in the review, given that reported raw scores for certain recovery outcomes still suggested an increase from baseline that participants could recover from. For example, in Gan et al. (2016), mean state anxiety for their three conditions during the stress task were* m_sedative_ *= 46.97,* m_stimulative_ *= 50.51, and* m_control_ *= 52.00, compared to baseline means scores of* m_sedative_ *= 38.80,* m_stimulative_ *= 38.09, and* m_control_ *= 36.17.* G*iven that the overall estimated effect of music listening on the recovery process of healthy individuals following laboratory stressors may be relatively modest, it becomes particularly important to ensure that a sufficient stress response is elicited, to provide a larger window of opportunity in which the effect of music listening may be exerted on participants’ recovery processes. We thus encourage future studies to adopt validated (variations of) well-known stress tasks, such as the TSST, SECPT, or CO2 stress task, which have been demonstrated to consistently elicit marked physiological and psychological stress-related responses in laboratory settings.”*

Gan, S. K. E., Lim, K. M. J., & Haw, Y. X. (2016). The relaxation effects of stimulative and sedative music on mathematics anxiety: A perception to physiology model. *Psychology of Music*, *44*(4), 730-741. https://doi.org/10.1177/0305735615590430

Labbé, E., Schmidt, N., Babin, J., & Pharr, M. (2007). Coping with stress: the effectiveness of different types of music. *Applied Psychophysiology and Biofeedback*, *32*, 163-168. https://doi.org/10.1007/s10484-007-9043-9

Scheufele, P. M. (2000). Effects of progressive relaxation and classical music on measurements of attention, relaxation, and stress responses. *Journal of Behavioral Medicine*, *23*, 207-228. https://doi.org/10.21236/ad1012237

**5. Page 11: “In our moderator analysis, we examined whether the effects of music listening on stress recovery were reliable across general (neuroendocrine, physiological, psychological) and specific outcome types.”**

**I am not sure whether the moderator analysis allows any claims about the reliability of the effects across outcome types. In theory, an effect could be highly reliable across many outcome types, while at the same time still being clearly stronger for some outcome types than for others (hence being moderated by them), right? Wouldn’t it be more correct to state that it was assessed to what extent the size of the effect on stress recovery depended on outcome type (or some equivalent formulation)? I am no expert on this issue, but I invite the authors to reconsider their wording.**

Thank you for your comment. Indeed, we utilized the term ‘reliable’ when in fact our intention was to assess the extent to which the size of the effect of music listening on stress recovery would differ, for example across outcome types. We have thus adjusted our wording to the following (page 12):

*“In our moderator analysis, we examined whether the effects of music listening on stress recovery* ***differed*** *across general (neuroendocrine, physiological, psychological) and specific outcome types.”*

**6. There is a type on page 15: Wisagreements 🡪 Disagreements**

Thank you for pointing this out. The typo (page 15) has been fixed.

*“****Disagreements*** *were resolved through face-to-face discussions, or through consultation with SG and KR when no consensus could be reached.”*

**7. As the authors rightly point out on page 6, stress recovery involves a process in which “changes that have occurred in response to a stressor revert to pre-stress baselines”. To quantify stress recovery, it therefore seems crucial to take individual pre-stress baseline levels into account.
To the reader, it does not readily become clear whether the effects derived from the studies included in the meta-analysis indeed reflect the extent to which stress levels “return to baseline”. From Table 4, the included studies seem to be a mix of 1) studies reporting differences in change scores with respect to pre-stress baseline levels and 2) studies reporting raw group differences in post-music stress levels. This may require some sort of disclaimer.**

**Could you please reflect on these analytical differences and their (possible) implications for the interpretation of your meta-analysis, in relation to the term “recovery”?**

Previous research has shown that following an acute stress reaction, all elevated physiological and psychological parameters will naturally revert to pre-stress baselines within 30-60 minutes (Hermans et al., 2014). As such, the most immediate proof of the effect of music listening on stress recovery would be to see whether listening to music would allow participants to reach their respective baseline levels sooner within time frame. Unfortunately, as we also point out in our Discussion, it is rare for studies to adopt a design where such changes are monitored, particularly through use of continuous measures. Instead, as you have rightly pointed out, studies either compare post-stress and post-manipulation change scores between conditions or compare post-manipulation raw group differences between music and comparable control conditions. The effects of music listening on stress recovery that we describe in our meta-analysis thus reflect how reactive participants’ stress recovery processes are when listening to music, rather than how soon participants recover, with the assumption that greater reactivity (e.g., larger decreases in heart rate) post-stressor also results in earlier returns to baseline.

Hermans, E. J., Henckens, M. J., Joëls, M., & Fernández, G. (2014). Dynamic adaptation of large-scale brain networks in response to acute stressors. *Trends in Neurosciences, 37*, 304-314. https://doi.org/10.1016/j.tins.2014.03.006

**8. On page 37-38 you write: “Khalfa et al. [55] reported that post-stressor cortisol decreased more rapidly for participants who listened to experimenter-selected classical music, compared with participants who sat in silence”**

**In Table 2 you write, when referring to this study: “Increase in post-stressor cortisol for music group significantly lower compared to control group (+)”.**

**These descriptions differ – could you please adapt the main text to match the (correct) description in the table?**

Thank you for pointing this out. The correct description was what we wrote in text. We have thus adjusted the description in Table 4 to:

*“Significant, rapid decrease in post-stressor cortisol in music group compared to control group (+).”*

**9. On page 40-41, you write: “While previous reviews suggest that music-based interventions may be moderately beneficial for stress-related outcomes, particularly in medical and therapeutic settings, our results suggest that the magnitude of this effect for healthy individuals may be more modest.”**

**While I largely agree with the contents of this paragraph (and with the further comments on this issue on page 46), I think the term “healthy individuals” (to label the category for which music is less effective for stress recovery) does not capture the essence of the differences between the different types of studies, and hence using this term may be a bit misleading.**

**As is stated further down the paragraph, the stress in studies conducted in medical and therapeutic settings likely has a more protracted time course, which does not directly have to do with the participants being (not) healthy. Furthermore, stress may differ in intensity between laboratory and medical real-life/settings, and the effectiveness of music may depend on the research setting as well.**

**It would be great if you could somewhat adapt the wording of this paragraph, to avoid the impression that the (non-) effectiveness of music depends on the participants being healthy. Rather, it seems more likely that several (interrelated) factors associated with the different research settings (e.g., type, intensity, and duration of stress) are driving these differences. You might e.g., use the term “healthy individuals under brief, experimentally induced stress” instead.**

Thank you for your comment. Indeed, the term “healthy individuals” may at times oversimplify the fact that the effect of music listening may differ based on differences in research settings. We have reworded the paragraph as follows:

*“The results of our review contrast those of previous meta-analyses, which underscore the relevance of music-based interventions for stress-reduction [11, 12]. While previous reviews suggest that music-based interventions may be moderately beneficial for stress-related outcomes, particularly in medical and therapeutic settings,* ***our results suggest that the magnitude of this effect outside of these settings, particularly for healthy individuals under acute, experimentally induced stress, may be more modest.*** *We presume that one of the principal reasons for this difference was our decision to exclude studies conducted in medical and therapeutic settings. In previous reviews, randomized controlled trials of the effects of music-based interventions within medical and therapeutic settings constituted a large portion of included studies: 67 of 79 (85%) studies in de Witte et al. [11], and 15 of 22 (68%) studies in Pelletier [12], making it more likely that overall effect sizes were derived from studies conducted within these settings.* ***Tentatively, the effects of music listening may be more prominent for the stress recovery of individuals in medical or therapeutic contexts, compared to that of individuals under acute stress in an experimental context****. Whereas the time course of stress responses and stress recovery in experimental settings can be considered relatively brief [25, 27, 41, 85], the time course of stress responses and stress recovery within medical and therapeutic settings may be significantly more protracted [13, 14]. Thus, within medical and therapeutic settings, music may be exerting its influence on neuroendocrine, physiological, and psychological processes that have been subjected to longer periods of strain [28, 108].”*

**Response to Reviewer #2**

Dear Reviewer,

Thank you for taking the time to review our manuscript. We appreciate the kind words you have mentioned on the execution of our review. More importantly, we sincerely appreciate the critical comments you have provided on our search strategy and adequacy of our approach. Below, we address your concerns in a point-by-point fashion and summarize the changes we have made to our manuscript based on your suggestions. You will see your comments in bold, our responses in regular text, and excerpts from our manuscript in italics (unless otherwise indicated):

**Major Concerns:**

**1) The authors identified 14 studies that are quite heterogenous in nature. I ask myself whether the approach of a meta-analysis is adequate for this rather small number of studies given this vast heterogeneity. Furthermore, not all studies were successful in stress induction. Wouldn't it be reasonable to exclude these studies from the analysis?**

We acknowledge that the relatively small number of heterogeneous studies may render the results of our meta-analysis less meaningful. We can thus understand if concerns are raised about whether a meta-analysis is the most appropriate approach to synthesize the available empirical evidence on the relationship between music listening and stress recovery.

As we state in the Limitations of our manuscript (page 49), we are aware that the small number of included studies makes it difficult to draw meaningful, substantial conclusions based on the results of the meta-analysis alone. For this reason, we have supplemented the quantitative synthesis of the meta-analysis with a more qualitative synthesis from a systematic review. We think this combined approach has yielded a more nuanced review, as the qualitative description of the included studies have helped provide more context to the results of our meta-analysis. We have reported the systematic review in our Results section on page 27.

Thus, we think our review is still valuable despite the small number of studies, as it provides not only a quantitative synthesis of available evidence, but also provides a qualitative description of the potential sources of heterogeneity that the meta-analysis could not account for.

Next, thank you for mentioning your concern over the inclusion of studies whose stress induction procedures were not successful. A similar point was raised by another reviewer. We thus agree that it is particularly important to further address the inclusion of studies with unsuccessful stress induction checks.

In the previous version of our manuscript, three studies were coded to have ‘unsuccessful’ stress induction checks. One of these studies (Scheufele, 2000) was erroneously coded, as the author did report a marked increase in heart rate following their stress induction procedure compared to baseline. Meanwhile, in the remaining two studies (Gan et al., 2016 & Labbé et al., 2007), the authors have hinted that their respective stress induction procedures were successful. However, we coded these as ‘unsuccessful’ since the statistical analyses comparing post-stressor and baseline values of their stress recovery outcomes were missing. We ultimately decided to still include these two studies in the meta-analysis given that the reported mean scores for certain stress recovery outcomes still suggested there was an increase from baseline from which participants could recover from. For example, in Gan et al. (2016), mean state anxiety for their three conditions during the stress task were *m_sedative_* = 46.97, *m_stimulative_* = 50.51, and *m_control_* = 52.00, compared to baseline means scores of *m_sedative_* = 38.80, *m_stimulative_* = 38.09, and *m_control_* = 36.17.

Based on this, we have thus updated the results of our analysis for the “stress induction checks” moderator (page 26), to reflect the change in coding for Scheufele (2000):

***“Stress induction checks.*** *There were no significant differences in the effects of music listening on stress recovery for studies with successful (*g *= 0.17301, 95% CI [-0.2635, 0.6155],* p *= .399625) and unsuccessful (*g *= 0.062361, 95% CI [-0.0894, 0.201.66],* p *= .115355) stress induction checks,* β1 *= -0.108257,* p *= .525661.”*

Next, we have added a paragraph to acknowledge the inclusion of studies with unsuccessful stress tasks in our Discussion (page 48):

*“Two studies with less successful stress induction procedures were still included in the review, given that reported raw scores for certain recovery outcomes still suggested an increase from baseline that participants could recover from. For example, in Gan et al. (2016), mean state anxiety for their three conditions during the stress task were* m_sedative_ *= 46.97,* m_stimulative_ *= 50.51, and* m_control_ *= 52.00, compared to baseline means scores of* m_sedative_ *= 38.80,* m_stimulative_ *= 38.09, and* m_control_ *= 36.17.* G*iven that the overall estimated effect of music listening on the recovery process of healthy individuals following laboratory stressors may be relatively modest, it becomes particularly important to ensure that a sufficient stress response is elicited, to provide a larger window of opportunity in which the effect of music listening may be exerted on participants’ recovery processes. We thus encourage future studies to adopt validated (variations of) well-known stress tasks, such as the TSST, SECPT, or CO2 stress task, which have been demonstrated to consistently elicit marked physiological and psychological stress-related responses in laboratory settings.”*

Gan, S. K. E., Lim, K. M. J., & Haw, Y. X. (2016). The relaxation effects of stimulative and sedative music on mathematics anxiety: A perception to physiology model. *Psychology of Music*, *44*(4), 730-741. https://doi.org/10.1177/0305735615590430

Labbé, E., Schmidt, N., Babin, J., & Pharr, M. (2007). Coping with stress: the effectiveness of different types of music. *Applied Psychophysiology and Biofeedback*, *32*, 163-168. https://doi.org/10.1007/s10484-007-9043-9

Scheufele, P. M. (2000). Effects of progressive relaxation and classical music on measurements of attention, relaxation, and stress responses. *Journal of Behavioral Medicine*, *23*, 207-228. https://doi.org/10.21236/ad1012237

**2) Concerning the search strategy, I wondered that PubMed was not included. Searching this data base might be useful as the number of studies identified is quite small.**

One of the goals of our review was to highlight the overall effect of music listening on stress recovery in healthy individuals. This meant excluding, for example, studies on the effects of music listening in the management of treatment anxiety or stress during pregnancy and labor. As we mention in the Introduction of our review (page 3), we reasoned that the nature of stressors in medical and therapeutic settings, along with their subsequent recovery processes, would be difficult to generalize to more daily settings.

From our experience, most studies on music listening published on the PubMed database reported experiments conducted within medical or therapeutic settings. Thus, when designing our search strategy, we made the decision to exclude the PubMed database from our search.

Despite this, based on your comment, we conducted an additional search in the PubMed database using the same search strategy listed in Appendix A. We limited the additional search to studies published until April 2021 to match our original search. This additional search returned 958 studies, but none of these studies met our inclusion criteria. Our search in PubMed thus resulted in no additional studies.

We have reported this additional search in our manuscript on page 10:

***“****The results of this primary search were supplemented with* ***three*** *additional electronic searches in the publication databases of Web of Science, PsycINFO,* ***and*** ***PubMed****. Appendix A provides a complete description of our search terms. Together, this first step resulted in* ***3124*** *articles.”*

We have also updated Figure 1 to include the addition of the PubMed search:

Information in the paragraph following Figure 1 has also been updated to reflect the additional search:

*“During this initial screening,* ***3008*** *articles were excluded. KA then scanned the reference lists of the* ***116*** *remaining articles for potentially relevant studies, resulting in an additional three articles. Together, this second step resulted in* ***119*** *full-text reports to be assessed for eligibility.”*

**3) Inclusion and exclusion criteria are not specifically justified. For example, in Figure 1 exclusion criteria are presented, e.g. 'no music presented after stressor'. This might be the reason why the study of Thoma et al. (2013) (https://pubmed.ncbi.nlm.nih.gov/23940541/) is not party of the review, although I consider it highly relevant in the context of music listening and stress reduction. Overall, I would describe and justify more in detail criteria for inclusion and exclusion of studies.**

Thank you for pointing this out. We agree that our inclusion and exclusion criteria could be better justified. As such, we have described our inclusion and exclusion criteria (pp. 10-11) more extensively, as follows:

*“Lastly, KA used the following criteria to assess full-text reports for eligibility:*

*First, to minimize between-study heterogeneity, and to ensure that included studies investigated the effects of music listening on stress recovery as precisely as possible, studies must employ an experimental design including stress induction, with random assignment of participants to experimental and control conditions. Quasi-experimental studies were included only when they incorporated a control or comparison group. Second, to ensure that included studies tested the immediate effect music listening may have on the stress recovery process, studies should compare music listening to silence or an auditory stimulus (e.g., white noise, audiobooks) following stress induction. Third, to demonstrate this effect, studies must include at least one measure of neuroendocrine (e.g., cortisol), physiological (e.g., heart rate, blood pressure), or psychological (e.g., subjective stress, positive and negative affect) stress recovery outcome. Fourth, given that stress reactivity and recovery responses differ between children and adults, and with consideration to the potential role of music in the prevention of stress-related diseases in adults, studies must include healthy, adult, human participants. Fifth, to improve the generalization of our results in the context of daily stress recovery, studies where stress recovery occurred within a medical or therapeutic context, such as a hospital or operating room, were excluded.”*

We agree that the findings of Thoma et al. (2013) are interesting and particularly relevant in the context of music listening and stress reduction. The experiment by Thoma and colleagues convincingly demonstrated that listening to music prior to a stressor resulted in a milder stress response compared to silence, which in turn resulted in a lower need for subsequent recovery. Although their finding speaks to the benefits of music listening in attenuating the stress response, their finding did not completely fit the scope of our review, which was the immediate effect of music listening on recovery from stress.

**4) Introduction: first paragraph: I really like the introduction to the topic, as the aspect of stress in daily life is emphasized. I wondered why the authors did not include ambulatory assessment/ecological momentary assessment studies in their review, as these studies have high ecological validity. I recommend to expand the review and to assess independently the evidence concerning controlled studies with high internal validity on the one hand and daily life studies with high ecological validity on the other hand.**

When we planned our review, we reasoned that focusing on experimental studies with high internal validity would allow us to examine the strongest available evidence on the presumed relationship between music listening and stress recovery. Furthermore, we hoped that, by focusing on experimental studies, between-study heterogeneity would thus be somewhat minimal – this was eventually not the case.

We agree that there is much to be gleaned from specifically investigating EMA studies on music listening and stress recovery, including further insight into interindividual differences when listening to music for the purpose of stress recovery, and how stress recovery outcomes may be influenced by music listening over time. Despite this, the inclusion of EMA studies in our review would have made it more difficult to determine the immediate effect of music listening on recovery from stress. Given the relatively lower control in EMA studies (e.g., the absence of a clear control condition), claims about causality may be trickier to draw from EMA studies compared to experiments. Furthermore, given that measurements occur outside of the laboratory, it becomes difficult to rule out the effects of contextual variables, particularly when they are not explicitly accounted for in the design of an EMA study. As such, we respectfully argue against the inclusion of EMA studies in our current review, given the stronger ‘causal’ evidence that may be derived from experimental studies, and because we agree that evidence from experimental and EMA studies should be assessed independently of each other due to differences in contextual factors.

**5) It may be my personal opinion, but I was irritated by the many instances the authors use the term 'beliefs', e.g., abstract 'given the popular and widespread belief'. My recommendation is to re-word this and acknowledge the empirical evidence underlying this statement.**

We apologize for the discomfort we have caused you as you reviewed our manuscript. We agree that, in principle, it is good to acknowledge available empirical evidence rather than labeling a statement a ‘belief’. We have thus adjusted the following instances of the term ‘belief’, starting with the Abstract:

*“Studies suggest that listening to music is beneficial for stress reduction. Thus, music listening stands to be a promising method to promote effective recovery from exposure to daily stressors.”*

On page 7:

*“Furthermore, studies have demonstrated that listening to music may influence mood [59, 60].”*

Finally, in our Conclusion:

*“Studies commonly suggest that listening to music may have a positive influence on stress recovery”*

**Minor Concerns:**

**Abstract: 'beneficial for stress' should be specified (beneficial for stress reduction)**

**Abstract: please report how many participants in total were included in the 14 studies
Abstract: please specify that (randomized)-controlled studies were included**

We have added the above suggestions to the Abstract:

*“Studies have suggested that listening to music may be* ***beneficial for stress reduction****. Thus, music listening stands to be a promising method to promote effective recovery from exposure to daily stressors.”*

*“As such, to clarify the current literature, we conducted a systematic review with meta-analysis of* ***randomized, controlled experimental studies*** *investigating the effects of music listening on stress recovery in healthy individuals.”*

*“In fourteen experimental studies, participants* ***(N = 706)*** *were first exposed to an acute laboratory stressor, following which they were either exposed to music or a control condition.”*

**Introduction: 'It is a popular and widespread opinion that music may be beneficial for
stress recovery [10]': I am not convinced that the citation is that adequate in this context. Levitin demonstrates in this book many instances for beneficial effects of music. I am not satisfied with labeling his statement as 'popular and widespread opinion'. Furthermore, I do not consider this book an optimal citation for this peer-reviewed journal article as there is a vast body of empirical evidence available.**

Thank you for mentioning this. We have rewritten the sentence and provided an alternative reference for it. Below is an excerpt from the paragraph (page 3), with the new sentence highlighted in bold:

*“Various activities have been proposed that may lead to better stress recovery, one among them being music listening.* ***Music listening may have a modulatory effect on the human stress response (Thoma et al., 2013).*** *Furthermore, given that music is readily available through online streaming services, music listening stands to be a time- and cost-effective method to facilitate daily stress recovery.”*

Thoma, M. V., La Marca, R., Brönnimann, R., Finkel, L., Ehlert, U., & Nater, U. M. (2013). The effect of music on the human stress response. *PLOS ONE, 8*, e70156. https://doi.org/10.1371/journal.pone.0070156

**Introduction l.60: My first thought was to question the necessity of this review given the fact that an extensive review was just published. Although, the authors justify their review in the ensuing paragraph, I would recommend to state immediately more clearly that the scope of the other review was different.**

Thank you for your suggestion. We agree that the urgency of our review could be stated earlier in the manuscript. We have restructured the two paragraphs as follows:

***“****Furthermore, given that music is readily available through online streaming services, music listening stands to be a time- and cost-effective method to facilitate daily stress recovery. Indeed, a recent meta-analysis of 104 randomized controlled trials on the effects of music concluded that music-based interventions have a positive impact on both physiological (d = .380, 95% CI [0.30–0.47]) and psychological (d = .545, 95% CI [0.43–0.66]) stress-related outcomes [11].* ***However, a large proportion of studies included in this meta-analysis were conducted in medical or therapeutic settings, and the included music-based interventions encompassed not only music listening but also music therapy. Thus, a more specific review to determine whether music listening alone is beneficial for the recovery of healthy individuals outside medical and therapeutic settings seemed justified.****”*

**Introduction l.78: Why not include studies demonstrating effects on cortisol?**

We have added studies demonstrating equivocal effects on cortisol to the sentence in question (page 4):

*“Indeed, studies on music listening and stress recovery in healthy individuals are equivocal: although music listening is considered beneficial for physiological stress recovery, several studies have reported no differences in heart rate, heart rate variability, respiration rate, or blood pressure, or* ***cortisol*** *recovery between participants who listened to music and those who either sat in silence or listened to an auditory control [18-****21****].”*

**l.133: I would not recommend to write 'smaller amounts of salivary alpha-amylase'. Rather, less activity of alpha-amylase.**

We agree that ‘less activity of alpha-amylase’ is more appropriate given what the outcome represents. We have replaced ‘amounts of salivary alpha-amylase’ accordingly (page 6):

*“This manifests as a restoration of parasympathetic activity, marked by a deceleration of heart rate and respiration rate, lower systolic and diastolic blood pressure, and* ***less activity of salivary alpha-amylase*** *[4, 29-32].”*

**l.153: Study 54 refers to an ambulatory assessment study – therefore, there was no control to silence or noise in a comparable manner to experimental studies. Please re-word.**

Thank you for pointing this out. We have adjusted the sentences accordingly (page 7):

*“For example, music listening has been associated with lower heart rate [49-51], systolic blood pressure [22, 50, 52], skin conductance [18, 20, 53, 54], and cortisol [55] compared to silence or an auditory control condition.* ***Similarly, participants who listened to music following stress demonstrated less activity of salivary alpha-amylase and lower cortisol compared to when music was listened to for other purposes [56].”***

**l.164: I do not agree with the statement that the candidate mechanism underlying beneficial effects of music has already been identified. I would rather prefer to see here a more comprehensive statement acknowledging that the exact underlying mechanisms remain to be elucidated and that different notions exist, e.g., literature by Koelsch…**

Thank you for your suggestion. It was not our intention to suggest that a definite mechanism underlying the beneficial effects of music listening on stress recovery has been identified. We agree that a more comprehensive statement would help convey this point more clearly. Following your suggestion, we have adjusted the paragraph accordingly (page 7-8):

*“The exact mechanisms underlying the effects of music listening on stress recovery remain to be elucidated. Music-evoked positive emotions are thought to be particularly beneficial for stress recovery, as they may help undo the unfavourable changes wrought by negative emotions during stress, ultimately aiding the stress recovery process (Tugade & Fredrickson, 2004). Alternatively, music-evoked emotions may promote a more robust, and thus more adaptive, stress response (Koelsch et al., 2016), which may be followed by an equally robust period of recovery. Next, it has been theorized that music may act as an anchor that draws attention away from post-stressor ruminative thoughts or negative affective states, thus preventing a lengthening of physiological activation, and facilitating a more regular stress recovery process (Baltazar et al., 2019; Radstaak et al., 2014). Finally, physiological rhythms in our body, such as respiration, cardiovascular activity, and electroencephalographic activity, may become fully or partially synchronized with rhythmical elements perceived in music (Ellis & Thayer, 2010; Trost et al., 2017). This rhythmic entrainment process is thought to occur via a bottom-up process that originates in the brainstem: salient musical features, such as tempo, pitch, and loudness, are continuously tracked by the brainstem, generating similar changes in ANS activity over time…”*

Baltazar, M., & Saarikallio, S. (2019). Strategies and mechanisms in musical affect self-regulation: A new model. *Musicae Scientiae*, *23*(2), 177-195. https://doi.org/10.1177/1029864917715061

Ellis, R. J., & Thayer, J. F. (2010). Music and autonomic nervous system (dys) function. *Music perception*, *27*(4), 317-326. https://doi.org/10.1525/mp.2010.27.4.317

Koelsch, S., Boehlig, A., Hohenadel, M., Nitsche, I., Bauer, K., & Sack, U. (2016). The impact of acute stress on hormones and cytokines and how their recovery is affected by music-evoked positive mood. *Scientific reports*, *6*(1), 1-11. https://doi.org/10.1038/srep23008

Radstaak, M., Geurts, S. A., Brosschot, J. F., & Kompier, M. A. (2014). Music and psychophysiological recovery from stress. *Psychosomatic medicine*, *76*(7), 529-537. doi: 10.1097/PSY.0000000000000094

Trost, W. J., Labbé, C., & Grandjean, D. (2017). Rhythmic entrainment as a musical affect induction mechanism. *Neuropsychologia*, *96*, 96-110. https://doi.org/10.1016/j.neuropsychologia.2017.01.004

Tugade, M. M., Fredrickson, B. L., & Barrett, L. F. (2004). Psychological resilience and positive emotional granularity: Examining the benefits of positive emotions on coping and health. *Journal of personality*, *72*(6), 1161-1190. https://doi.org/10.1111/j.1467-6494.2004.00294.x

**l.274: IgA is named as outcome measure but has not been introduced. As it is an immune marker, the introduction should contain some information on interactions among stress and immune system.**

Thank you for the reminder. We have added an additional sentence in the Introduction to present salivary IgA as a marker for stress (page 5):

*“This process enables rapid, non-genomic effects that sustain ANS-mediated changes for the duration of the stressor, while suppressing immune system function [33-35].* ***This suppression is visible through lower concentrations of immunoglobulins, such as salivary immunoglobulin-A (s-IgA; Chojnowska et al., 2021).****”*

Chojnowska, S., Ptaszyńska-Sarosiek, I., Kępka, A., Knaś, M., & Waszkiewicz, N. (2021). Salivary biomarkers of stress, anxiety, and depression. *Journal of Clinical Medicine*, *10*(3), 517. https://doi.org/10.3390/jcm10030517

**l.310: some typos need revision**

Thank you for pointing this out. The typos have been revised (page 15):

*“****Disagreements*** *were resolved through face-to-face discussions, or through consultation with SG and KR when no consensus could be reached.”*

**l.425: Can you please indicate the range of music duration? As there is literature available on the effects of different music durations on beneficial effects, I assume that the range was very limited among these studies. Therefore, I would not state that duration is not important. Rather, that the range in experimental studies is not vast enough to allow for meaningful comparisons.**

We have added the range of music duration in our report of the moderator analyses (page 26):

*“****Duration of music.*** *There was no evidence that the effect of music listening on stress recovery may differ depending on how long participants were exposed to music, β1 = -0.005, p = .870* ***(*range_duration_ *= 2 – 45 minutes)****.”*
