# Supplementary material for: Music listening and stress recovery in healthy individuals: A systematic review with meta-analysis of experimental studies
Source: PLoS One. 2022 Jun 17;17(6):e0270031. doi: 10.1371/journal.pone.0270031 (PMC9205498; doi:10.1371/journal.pone.0270031)
Supplement: Attachment — Submitted filename: ma20220426_rebuttal_PLOS.docx [file pone.0270031.s005.docx]

Dear Prof. Dr. Nater,

Thank you for your e-mail in response to our revised submission to PLOS ONE [PONE-D-21-22806R1], in which you invite us to consider a few additional issues raised by one of the reviewers. We have carefully addressed these issues and adjusted our manuscript accordingly. Our responses to each reviewer have been provided in a point-by-point manner below.

We hope this second revision to our manuscript will completely meet PLOS ONE’s rigorous publication criteria. We look forward to hearing from you.

Warm regards,

Krisna Adiasto, MSc.

On behalf of all authors.

**Response to Reviewer #1**

Dear Dr. Majdandžić,

Thank you for taking the time to review our revised manuscript. Given your expertise in the effects of music listening on stress and wound healing, we are grateful that you have recommended the manuscript for publication in its current form.

**Response to Reviewer #2**

Dear Reviewer,

Thank you for taking the time to review our revised manuscript. We once again appreciate your kind words on our work and are happy to read that you consider the manuscript to be stronger in its current form. Below, we address the additional issues you have raised and summarize the changes we have consequently made to our manuscript in a point-by-point fashion. You will see your comments in bold, our responses in regular text, and excerpts from our manuscript in italics (unless otherwise indicated):

**1) When reading your response letter I considered it an excellent idea to combine both systematic review and meta-analytic approach. However, when I read the manuscript, I felt it overloaded the paper. Furthermore, I did not really understand, why the number of included studies varies among these two approaches (14 vs. 17 studies).**

**My suggestion would be to either start with describing the review approach and then calculate the overall meta-analytic effect or to move the systematic review to the Appendix (after having adjusted the analysis to the same number of studies included).**

Thank you for your suggestion. In one of the earliest drafts of our review, we chose to present the systematic review section prior to the meta-analysis. Thus, the meta-analysis served to quantify the extensive qualitative evidence we presented in the systematic review. In line with this approach, we initially decided that studies which did not (or could not) provide the necessary means and standard deviations to estimate effect sizes (in our case, Hedge’s *g*) would be excluded from the meta-analysis. However, since means and standard deviations are less relevant to a qualitative review, we decided that studies without means and standard deviations could still be included in the systematic review section instead of being excluded completely, provided they met the rest of our inclusion criteria. As such, in the previous version of our manuscript, 17 studies were part of the systematic review section, while only 14 of those studies were part of the meta-analysis section.

In the *previous* version of our manuscript, we attempted to briefly explain this through the following information:

*“Based on these criteria, the final sample for the systematic review portion of our review consisted of 17 studies. Finally, for studies to be included in the meta-analysis portion of our review, means and standard deviations of stress recovery outcomes following stressor cessation must be available. Corresponding authors were contacted when this information was not available. When authors did not or could not provide the required information (e.g., due to data no longer being accessible), the outcome was dropped from the meta-analysis. Thus, following attempts to obtain missing information, the final sample for the meta-analysis portion of our review consisted of 14 studies.”*

In the *current* version of our manuscript, the meta-analysis section is currently presented first, with the systematic review section painting a more detailed picture about the methodological heterogeneity between included studies. We understand that an extensive qualitative portion which directly follows a straight-forward quantitative synthesis can feel somewhat overwhelming. Thus, we have decided to follow your suggestion and moved the bulk of the systematic review portion to the Appendix of our manuscript.

To accommodate this change. we have adjusted the paragraph in our inclusion criteria as follows (page 11):

*“Finally, for the purpose of the meta-analysis, means and standard deviations of stress recovery outcomes following stressor cessation must be available. Corresponding authors were contacted when this information was not available. When authors did not or could not provide the required information (e.g., due to data no longer being accessible), outcomes were dropped from the meta-analysis. Following attempts to obtain missing information, the final sample for our review consisted of 14 studies.”*

Furthermore, we have added the following paragraph after the results of our moderator analyses, to direct readers’ attention towards the systematic review in the Appendix (page 26):

*“To further illustrate the methodological heterogeneity among experimental studies on the effect of music listening on stress recovery, we provide a more extensive, qualitative overview of the included studies in Appendix C. A summary of this overview is presented in Table 4.”*

**2) Thank you for describing in more detail how you operationalized unsuccessful stress induction. I am not entirely convinced by your approach. For example, you provide mean statistics for two studies and conclude that the mean difference represents a successful stress induction. At least, I would expect a citation backing this up or a statistical test considering mean and standard deviation. I wondered if it was more appropriate to distinguish successful from unsuccessful and (third category) not reported. As for now, I would still argue to include only those studies with successful stress reduction (especially given the unequal ratio that limits comparisons anyways).**

Thank you for mentioning your concern. In the two studies we have labelled ‘unsuccessful’ with regards to their stress induction procedures, the authors do hint at the success of their stressors in their respective manuscripts. However, since this success was not explicitly reported (e.g., through a comparison between baseline and post-stressor outcomes), we ultimately decided to label these as ‘unsuccessful.’

For example, in Gan et al. (2015), the authors cite a statistically significant paired-samples t-test comparing pre-stressor and post-music math anxiety in their no-music control group (only) as evidence that their stress induction procedure was successful for all their conditions. Fortunately, Gan et al. (2015) have reported pre- and post-stressor (i.e., pre-music) means and standard deviations for all our outcomes of interest. Thus, we were able to conduct our own paired-samples t-tests using pooled standard deviations, assuming a correlation of 0.5 between pre- and post-stressor outcome measures (Estrada et al., 2018). Our own t-tests indeed demonstrate that there is a significant increase in stress from which participants can recover from.

We were not able to employ a similar method for Labbé et al. (2007), as the authors did not explicitly report pre- and post-stressor means and standard deviations for our outcomes of interest. However, Labbé et al. (2007) presented the results of several F-tests with significant effects of time (under stress/pre-music vs. post-music) for all their conditions. Though these tests are not as accurate as a baseline vs. post-stressor comparison to evaluate the effects of stress induction procedures, we considered it plausible that a stress reaction had indeed occurred.

With these considerations in mind, we agree that labeling both studies as ‘unsuccessful’, with regards to their stress induction procedures, may not be the most correct decision. Thus, we have decided to follow your suggestion and change their coding to ‘unreported’ instead – in the sense that what the studies reported were not an explicit test of their stress induction procedures.

Our final consideration to keep these two studies with unreported stress induction procedures in the meta-analysis is that the estimated cumulative effect size *excluding* the two studies (i.e., comprising studies with successful stress induction only), *g* = 0.173, 95% CI [-0.26, 0.61], *p* = .399 (page 25):

“***Stress induction checks****. There were no significant differences in the effects of music listening on stress recovery for studies with successful* ***(*g *= 0.173, 95% CI [-0.26, 0.61],* p *= .399)*** *and unreported (*g *= 0.062, 95% CI [-0.08, 0.20],* p *= .115) stress induction checks,* β_1_ *= -0.108,* p *= .661.”*

…does not significantly differ in magnitude or significance with the overall cumulative effect size *including* the two studies (i.e., comprising studies with successful and unreported stress induction), *g* = 0.15, 95% CI [-0.21, 0.52], *p* = 0.374 (page 23):

*“Based on a meta-regression with RVE, the estimated overall effect of music listening on stress recovery was* **g *= 0.15, 95% CI [-0.21, 0.52],* t*(13) = 0.92,* p *= 0.374.****”*

With regards to this matter, we have updated our discussion on page 38:

*“Two studies with unreported stress induction procedures were still included in the review [17,84], as reported means for certain recovery outcomes still suggested an increase from baseline that participants could recover from. For example, with the information reported in Gan et al. [84], assuming a correlation of 0.5 between baseline and post-stressor measures of state anxiety, we estimated that their stress induction procedure elicited a significant increase in state anxiety in their sedative music (t(34) = 5.87, p < .001, mdiff = 8.17, SDdiff = 8.24), stimulative music (t(34) = 8.21, p < .001, mdiff = 12.42, SDdiff = 8.95), and control (t(34) = 13.15, p < .001, mdiff = 15.83, SDdiff = 7.12) conditions. As the overall estimated effect of music listening on the recovery process of healthy individuals following laboratory stressors may be relatively modest, it becomes particularly important to ensure that a sufficient stress response is elicited, to provide a larger window of opportunity in which the effect of music listening may be exerted on participants’ recovery processes. We thus encourage future studies to adopt validated, (variations of) well-known stress tasks, such as the TSST [109], SECPT [110], or CO2 stress task [111], which have been demonstrated to consistently elicit marked physiological and psychological stress-related responses in laboratory settings. Furthermore, we remind future studies to candidly report the results of their stress induction procedures to facilitate subsequent meta-syntheses of the effects of music listening on stress recovery.”*

Estrada, E., Ferrer, E., & Pardo, A. (2019). Statistics for evaluating pre-post change: Relation between change in the distribution center and change in the individual scores. *Frontiers in psychology*, *9*, 2696.

Gan, S. K. E., Lim, K. M. J., & Haw, Y. X. (2016). The relaxation effects of stimulative and sedative music on mathematics anxiety: A perception to physiology model. *Psychology of Music*, *44*(4), 730-741.

Labbé, E., Schmidt, N., Babin, J., & Pharr, M. (2007). Coping with stress: the effectiveness of different types of music. *Applied psychophysiology and biofeedback*, *32*(3), 163-168.

**3) Thank you for describing in more detail your inclusion criteria. As they do not cover 'music should have been played after the stressor', I still argue that the Thoma Paper should be included. Therefore, please change the inclusion criteria accordingly or include the paper. If you adjust the inclusion criteria, I would recommend to refer to the Thoma Paper in the discussion as time of intervention (before, during or after stressor) might be an important modulator.**

Thank you for pointing this out. We have revised our inclusion criteria to make it clearer that we elected to focus on studies looking at the effects of music listening on stress recovery after a stressor (page 11):

*“Second, studies should compare music listening to silence or an auditory stimulus (e.g., white noise, audiobooks). To ensure that included studies tested the immediate effect of music listening on stress recovery, exposure to music, silence, or auditory stimuli must occur after the stress induction procedure.”*

Next, given our focus, we agree that the timing of the music intervention may be an important moderator of the effects of music listening on stress recovery. Thus, we have added the following paragraph to our Discussion, referring to several studies investigating the effects of music listening at different timings, including the Thoma et al. (2013) paper (page 38-39):

*“As the current review focused on the effects of music listening after a stressor, studies where music was played before or during a stressor were omitted from our analyses. However, several studies suggest that the timing at which music is played (i.e., before, during, or after a stressor) may influence its effects on stress recovery. For example, in Burns et al. [48], participants who listened to classical music while anticipating a stressful task exhibited lower post-music heart rate compared to participants who anticipated the stressor in silence. Similarly, concentrations of salivary cortisol were lower for participants who watched a stressful visual stimulus while listening to music compared to those who watched the same stimulus without music [112]. Together, these findings hint that, when listened during a stressor, music may attenuate cortisol responses [9,113], thus reducing the subsequent need for recovery. On the other hand, Thoma et al. [9] reported that participants who listened to music prior to a stressor exhibited higher post-stressor cortisol compared to participants who listened to an audio control. Interestingly, despite the stronger stress response, Thoma et al. [9] noted a trend for quicker ANS recovery among participants who listened to music, particularly with regards to salivary alpha-amylase activity. This pattern of findings is consistent with the notion forwarded by Koelsch et al. [61], in that music listening may promote a more adaptive stress response, thus facilitating subsequent stress recovery processes. To date, research on timing differences in the context of music listening and stress recovery is scarce. Thus, future studies could further examine the influence of such timing differences to better understand their role in the relationship between music listening and stress recovery.”*

**4) I am sorry to read that you decided against EMA studies as I consider it a huge strength to combine both experimental and EMA evidence. I am not convinced that these two approaches should be studied separately, as they complement each other in a meaningful way. Also, I believe that including EMA studies would shed light on the heterogeneity as you have multiple time points and multiple contextual factors being repeatedly assessed over time. Nevertheless, I accept your choice here, but recommend to acknowledge EMA studies in the discussion (or outlook). Particularly as you describe in the introduction that music is so easily available, studying the mechanisms in daily life seems to be timely.**

Thank you for your explanation, and we apologize to not have been able to account for EMA studies at the present time. We have followed your suggestion and acknowledged the value of EMA studies in our Discussion section (page 39):

*“Given the pervasiveness of stress, Ecological Momentary Assessment (EMA) studies may provide a more intimate outlook on the dynamics of daily music listening behaviour, particularly for the purpose of stress recovery. For example, through an ambulatory assessment study, Linnemann et al. (38) revealed that music produced the most notable reductions in physiological and psychological stress outcomes when it was listened to for the purpose of ‘relaxation’, compared to other reasons such as ‘distraction’, ‘activation’, and ‘reducing boredom’. Indeed, given their high ecological validity, EMA studies may provide further insight into important contextual variables in the relationship between music listening and stress recovery. For example, in an EMA study, listening to music in the presence of others was related to decreased subjective stress, attenuated cortisol secretion, and higher activity of salivary alpha-amylase (55). Furthermore, physiological responses to music may co-vary between members of a dyad when music is listened to by couples (114). Thus, given the benefits of EMA studies, we invite future studies to continue exploring the dynamics and contextual factors of music listening behaviour for stress recovery in daily life.”*

**5) Please omit the following sentence from the manuscript as I am afraid that it does not reflect the findings on alpha-amylase appropriately.**

**Similarly, participants who listened to music following stress demonstrated less activity**

**of salivary alpha-amylase and lower cortisol compared to when music was listened to for other purposes [56].”**

Our apologies for not appropriately conveying the effects of music listening on salivary alpha-amylase. The sentence in question has been deleted. The paragraph now reads:

*“**For example, music listening has been associated with lower heart rate [48–50], systolic blood pressure [21,49,51], skin conductance [17,19,52,53], and cortisol [54,55] compared to silence or an auditory control condition. Furthermore, music listening has been associated with higher parasympathetic activity [56] compared to silence [3,37]. Together, these findings suggest that music listening may generate beneficial changes in ANS and HPA axis activity that should be conducive to the stress recovery process [27,57,58].”*
